# Treatment of Port‐Wine Birthmarks Using a Large‐Spot, Variable Sequenced, Long‐Pulsed KTP‐Laser—A Retrospective Analysis

**DOI:** 10.1111/jocd.70152

**Published:** 2025-03-28

**Authors:** Lynhda Nguyen, Stefan W. Schneider, Katharina Herberger

**Affiliations:** ^1^ Department of Laser and Aesthetics, Department of Dermatology and Venereology University Medical Center Hamburg‐Eppendorf Hamburg Germany; ^2^ Department of Dermatology and Venereology University Medical Center Hamburg‐Eppendorf Hamburg Germany

**Keywords:** KTP Laser, port‐wine stain, pulsed dye laser, vascular Lesion

## Abstract

**Background:**

Pulsed‐dye lasers (PDLs) are currently regarded as the gold standard for treating port‐wine birthmarks (PWBs). Recently, the large‐spot, variable‐sequenced, long‐pulsed potassium titanyl phosphate laser (KTP) has been introduced as a promising alternative for addressing dermatologic vascular lesions. Despite its growing use, data on the efficacy and safety of this technology in treating PWBs over an extended period remain limited.

**Aims:**

To evaluate the efficacy and safety of a large‐spot, variable‐sequenced, long‐pulsed KTP in the treatment of PWBs over 3 years.

**Methods:**

A retrospective analysis was conducted on PWB patients treated with the KTP between October 2021 and October 2024. Treatment outcomes were assessed using colorimetric analysis based on the CIE L*a*b* color space system and by measuring the surface area of the lesions. All adverse events (AEs) were documented.

**Results:**

A total of 69 patients were included, with a mean age of 36.5 ± 15.6 years. 59.4% were female. Colorimetric analysis revealed an average lightening of PWBs by 75.7% ± 36.1%. Additionally, the surface area of the lesions decreased by 25.3% ± 24.4%. No severe AEs, such as post‐inflammatory hyperpigmentation or scarring, were observed.

**Conclusion:**

This study highlights the potential of large‐spot, variable‐pulsed KTP as a safe and effective alternative for treating PWBs, demonstrating significant lesion lightening and surface area reduction. With no severe AEs observed, the findings suggest that this technology could complement or serve as an alternative to PDLs. Further prospective studies are warranted to optimize treatment protocols and evaluate long‐term outcomes compared to PDL and other vascular treatment options.

## 1 Introduction

Port‐wine birthmarks (PWBs) are congenital vascular malformations, caused by ectatic capillaries and postcapillary venules [1, 2]. PWBs affect approximately 0.3%–0.5% of the population and are most commonly found on the head and neck region, though they can occur on any part of the body [3]. Initially, PWBs typically present as sharply demarcated erythematous to livid macules [4]. Over time, these lesions may darken and thicken, potentially developing hypertrophic or nodular changes [4]. The prominent and visible nature of PWBs can lead to substantial psychological and emotional challenges for affected individuals [5, 6]. Laser treatments have been shown to improve both the physical appearance of PWBs and the quality of life for patients [7, 8, 9]. This underscores the importance of effective treatment strategies that address both the cosmetic and psychosocial impacts of this condition.

Several laser‐ and light‐based technologies are available for treating PWBs [3, 10]. Among these, pulsed‐dye lasers (PDLs) are considered the gold standard. The PDL operates on the principle of selective photothermolysis [11]. Here, the 585 or 595 nm wavelength is selectively absorbed by hemoglobin and oxyhemoglobin while melanin absorption remains relatively low [12]. The PDL is one of the first lasers working with this principle in mind and has remained a cornerstone in the management of vascular lesions [13].

In recent years, the 532 nm large‐spot, variable‐sequenced, long‐pulsed potassium‐titanyl‐phosphate laser (KTP) has emerged as a potential alternative for treating PWBs and other vascular diseases. Early studies comparing large‐spot KTPs with PDLs have demonstrated promising efficacy and safety profiles [14, 15]. However, data on outcomes in patients with PWBs over an extended period are still lacking.

This study aimed to address these gaps by evaluating the efficacy and safety of the large‐spot KTP in the treatment of PWBs over years. The findings are intended to provide deeper insights into the clinical utility of the KTP and its potential role in optimizing treatment approaches for PWBs.

## 2 Material and Methods

### 2.1 Study Design

This study was conducted as a retrospective analysis including patients from October 2021 to October 2024. Data were collected from patients treated at the Department of Laser and Aesthetics at the University Medical‐Center Hamburg‐Eppendorf. Inclusion criteria encompassed PWB patients who underwent treatment with a large‐spot, variable‐pulse KTP and had corresponding three‐dimensional (3D) photo documentation available. Additionally, patients who participated in a previous prospective study and received further KTP treatments after its completion were also included [14].

### 2.2 Treatment Protocol

For treatment, a large‐spot, variable pulsed 532 nm KTP/Nd:YAG laser (DermaV, Cynosure Lutronic, Hamburg, Germany) was used. Parameters were chosen to achieve a subpurpuric reaction (Supporting Information S1). Two passes were applied to address the lesions at different depths. Sessions were scheduled at intervals of 6–12 weeks. For detailed settings, see Table 1.

**TABLE 1:** KTP parameters chosen for treating port‐wine birthmarks. The cryogen cooling pre‐, during, and post‐treatment was adjustable.

Passes	Spot size [mm]	Pulse length [ms]	Fluence [J/cm^2^]	Cryogen cooling [ms]
First pass	10–12	10, submilli subpulses	6–11	15–15—10
Second pass	10–12	10, submicro subpulses	6–11	15–15—10

### 2.3 Data Collection

The following data were collected: patient age, gender, Fitzpatrick skin type, location of PWBs, prior treatments, number of KTP sessions, and changes in the erythema and size of PWBs.

### 2.4 Colorimetric Assessment and Surface Measurement

Standardized photographs were taken using a 3D imaging system (Vectra, Canfield Scientific Inc., Bielefeld, Germany). Erythema changes were assessed with the CIE L*a*b* color space system (Commission Internationale de l'Éclairage), a numerical model aligned with human color perception [16]. L* represents lightness (gray scale), a* quantifies the green (−a) to red (+a) axis, and b* indicates the blue (−b) to yellow (+b) axis. For each PWB, multiple areas were measured at baseline and 6–12 weeks post‐treatment. The overall improvement in lesion coloration was quantified by calculating the ΔE value, which reflects the difference between the erythema of the PWB and the surrounding non‐affected skin. The calculation was adapted from the equation proposed by Tremaine et al. [17] Computer‐assisted tracing was applied to measure identical spots. Area reduction was assessed at 6–12 weeks post‐treatment compared to baseline using the VAM software (Canfield Scientific Inc., Bielefeld, Germany).

### 2.5 Adverse Events

In case of adverse events (AEs), written and photo documentation was done.

### 2.6 Statistical Analysis

Statistical analysis was conducted using Microsoft Excel (version 16.56) and SPSS Statistics (version 29.0.2.0, IBM). Unless stated otherwise, paired *t*‐tests were used to compare means before and after treatment. The Mann–Whitney‐Wilcoxon test was applied to evaluate changes in Δ*E* following KTP treatment. Descriptive data are presented as means with standard deviations (SD) and ranges (minimum–maximum). A *p*‐value of < 0.05 was considered statistically significant.

## 3 Results

### 3.1 Baseline Characteristics

A total of 69 patients were included in the study, with a mean age of 36.5 ± 15.6 years (2–71). Of these, 59.4% were female. The majority had Fitzpatrick skin type II (66.7%). Most PWBs (82.6%) were located on the face, with an average surface area of 28.8 ± 40.7 cm^2^ (0.2–234.3). Before undergoing KTP treatment, 59 patients (85.5%) had received prior treatments. Among these, 84.1% of patients had undergone PDL treatments, averaging 6.2 ± 5.5 sessions (1–26). Additionally, 7.2% received Nd:YAG laser treatments, with an average of 1.6 ± 0.9 sessions (1–3). In this study, patients received 5.6 ± 3.4 KTP sessions (1–15). Further details are outlined in Table 2.

**TABLE 2:** Baseline characteristics of included patients.

Baseline characteristics	Total
Patients [*n*]	69
Mean age [years] (SD, range)	36.5 ± 15.6 (2–71)
Male/female [*n*]	28/41
Fitzpatrick skin type [*n* (%)]	
I	7 (10.1)
II	46 (66.7)
III	10 (14.5)
IV	5 (7.2)
V	1 (1.5)
VI	0 (0)
Mean surface area [cm^2^] (SD, range)	28.8 ± 40.7 (0.2–234.3)
Location of port‐wine birthmarks [*n* (%)]	
Face	57 (82.6)
Neck	5 (7.2)
Trunk	2 (2.9)
Upper arm	3 (4.3)
Forearm	2 (2.9)
Thighs	1 (1.4)
Lower leg	2 (2.9)
Pretreatments [*n* (%)]	
Pulsed‐dye laser	58 (84.1)
Nd:YAG laser	5 (7.2)
CO_2_ laser	1 (1.4)
Argon laser	1 (1.4)
Radium treatment	1 (1.4)
Surgical excision	1 (1.4)

### 3.2 Erythema Assessment

**FIGURE 1:**
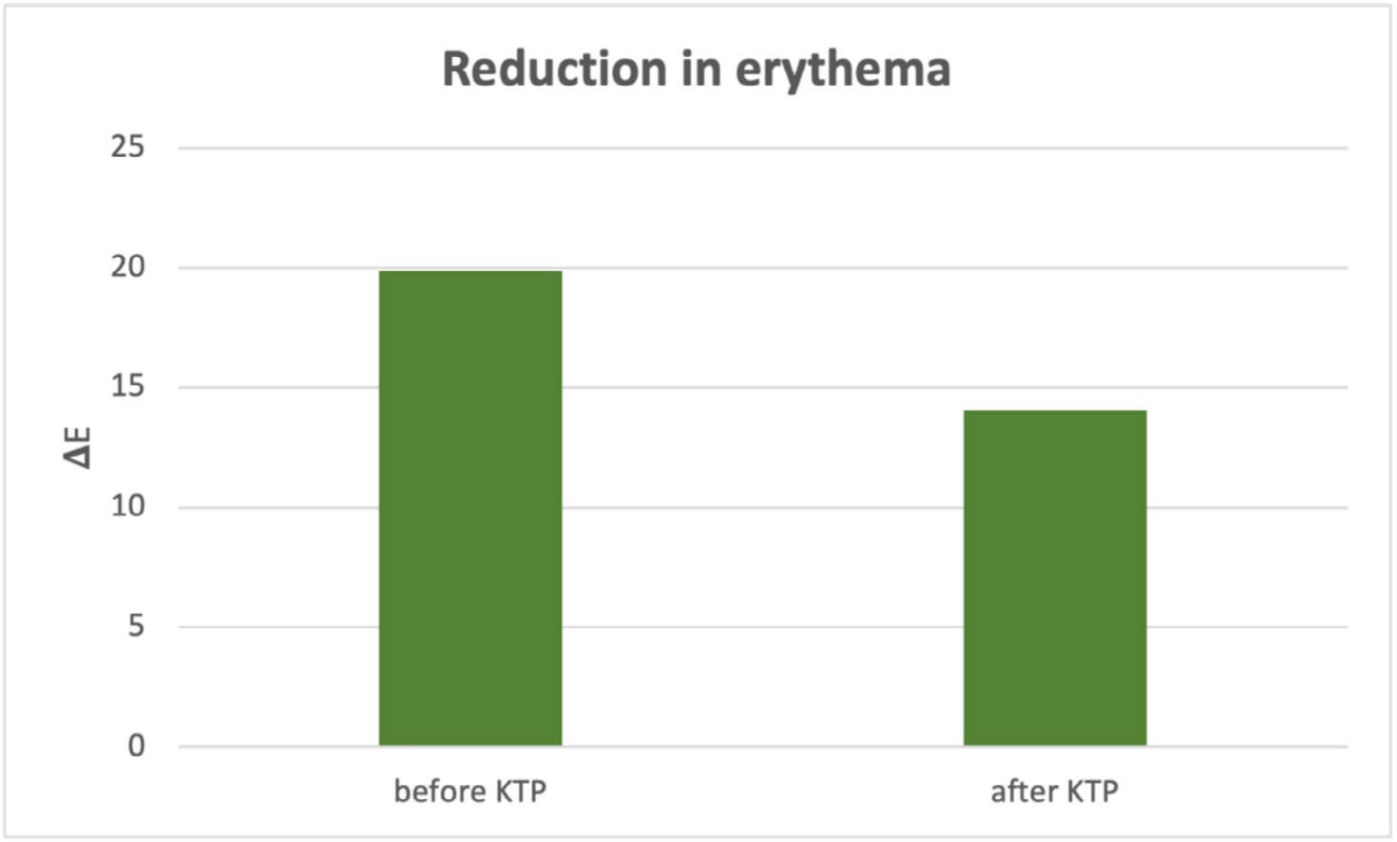
Reduction of erythema in port‐wine birthmarks (PWB) after KTP treatment. Based on the CIEL*a*b* color space system, △*E* was determined as the erythema difference between PWBs and surrounding non‐lesional skin.

**FIGURE 2:**
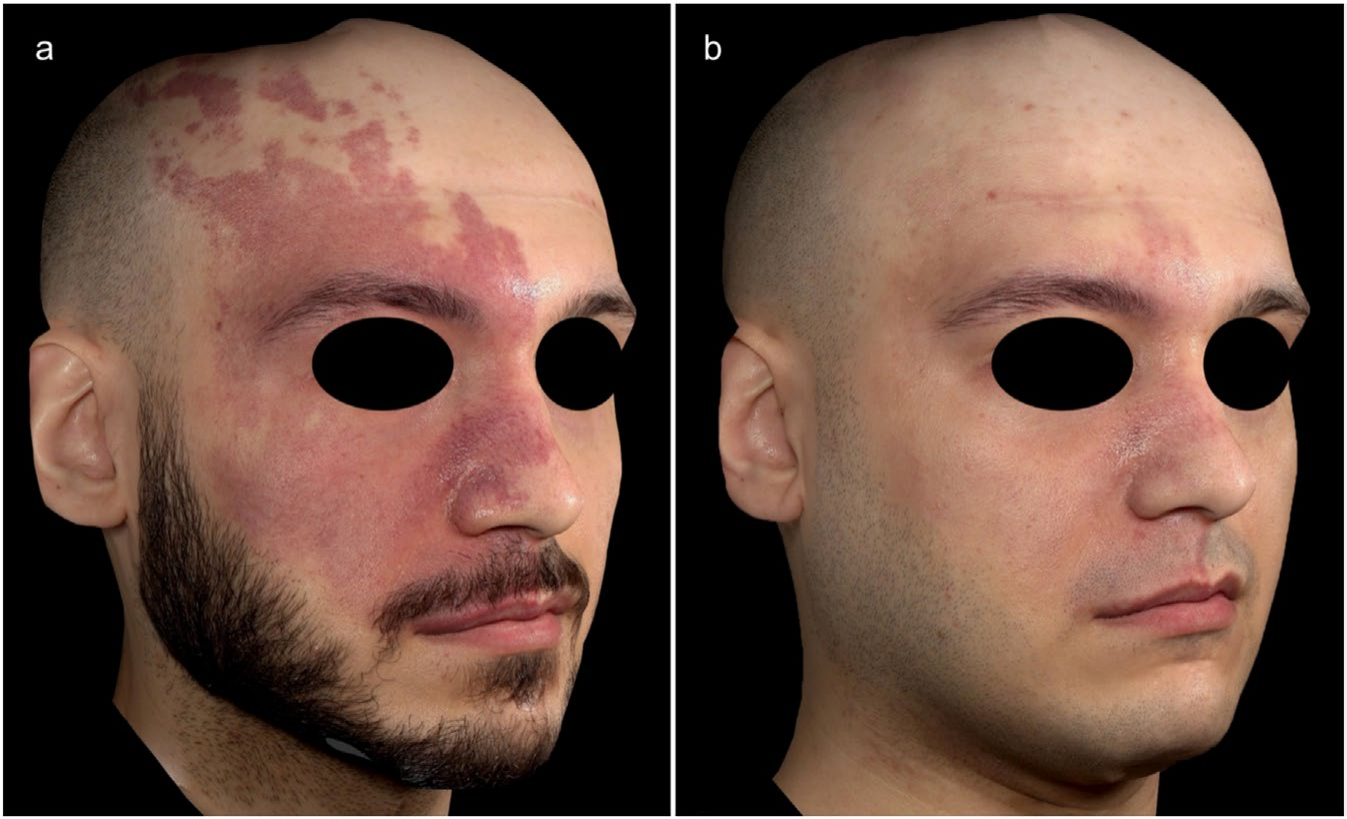
A 29‐year‐old patient (a) prior to treatment and (b) following 12 KTP sessions. The patient had previously undergone one pulsed‐dye laser session.

**FIGURE 3:**
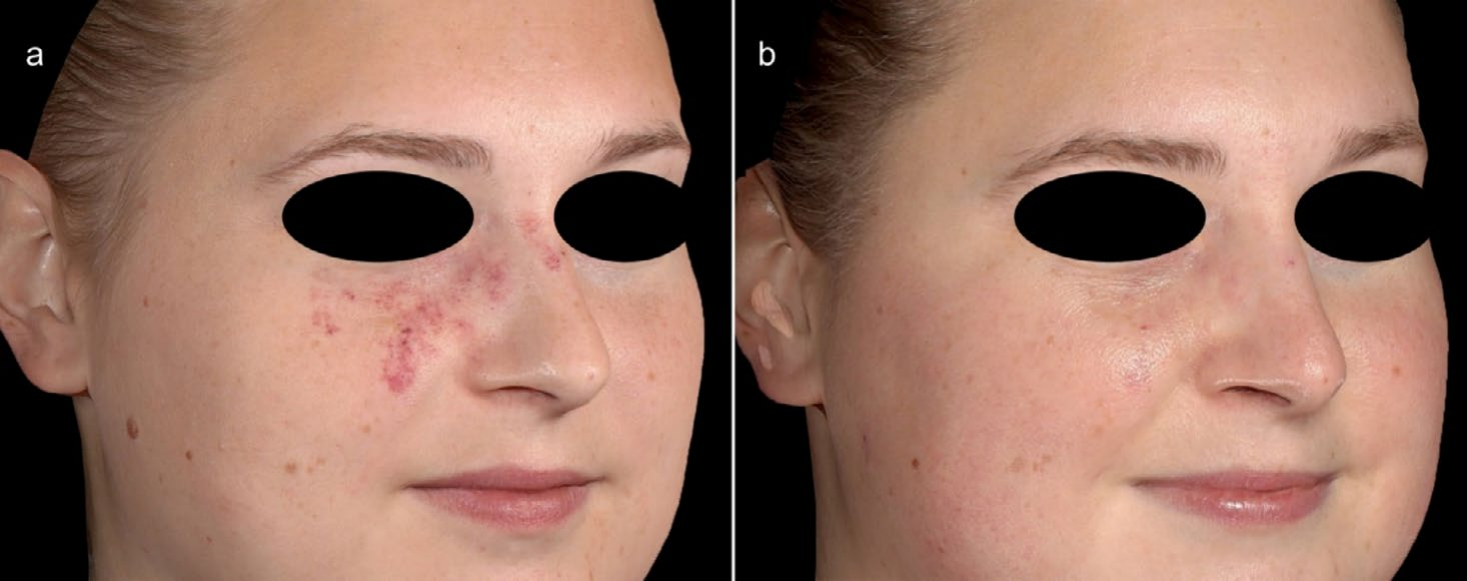
A 27‐year‐old patient (a) before and (b) after three sessions with a KTP. Prior to this, she had received 10 pulsed‐dye laser treatments.

Colorimetric analysis showed a △*E* value of 19.9 ± 9.4 (4.1–50.9) between non‐lesional skin and PWBs at baseline. After an average of 5.6 ± 3.4 KTP sessions, △*E* significantly decreased to 14.1 ± 8.3 (1.8–54.0; *p* < 0.05), indicating a measurable improvement in lesion clearance (Figure 1). This corresponded to an erythema reduction of 75.7% ± 36.1% (12.7 to −63.7). Figures 2 and 3 indicate examples of patients with PWBs before and after KTP treatments.

### 3.3 Surface Area Assessment

Surface area significantly decreased from 28.1 ± 40.0 cm^2^ (0.2–234.3) to 22.7 ± 34.6 cm^2^ (0.1–214.4; *p* < 0.05). This corresponds to a reduction of 25.3% ± 24.4% (−15.1–95.7).

### 3.4 Side Effects

All patients experienced erythema and mild swelling immediately following treatment, which resolved within a few days. Three patients (4.3%) developed crusting that healed within a few days. One patient (1.4%) experienced significant swelling lasting 3 days, requiring time off work. No instances of blistering, scarring, or postinflammatory hyperpigmentation (PIH) were observed.

## 4 Discussion

This study is the first to examine the potential of a large‐spot, variable‐sequenced, long‐pulsed KTP for achieving lesion clearance and evaluating safety in the treatment of PWBs over a period of more than 3 years. Our findings revealed significant lightening of PWBs based on objective colorimetric analysis, alongside a notable decrease in size.

Treating PWBs in adulthood is known to pose greater challenges compared to initiating therapy during infancy, as earlier treatment benefits from thinner skin and less scattering [18, 19]. This distinction makes it particularly important to evaluate effective treatments for adult patients, who often experience a great psychological and social burden from visible PWBs and thus seek treatment [5, 6].

Moreover, the majority of patients in this study had previously undergone PDL sessions. A central focus of this research was to determine whether subsequent treatments with a large‐spot, long‐pulsed KTP could provide additional or distinct improvements. The findings suggest that the KTP shows significant potential as either an alternative or complementary treatment for patients seeking further improvement after PDL therapy. These results highlight the potential of KTPs to expand therapeutic options and enhance outcomes for individuals with PWBs. Notably, complete clearance with PDL therapy remains rare, with approximately 20% of lesions showing no response at all [20]. Additionally, lesion re‐darkening due to revascularization poses a significant limitation, with up to 50% of patients reporting PWB re‐darkening as early as 5 years post‐treatment [21, 22]. Given these challenges, the role of KTPs in addressing PDL‐refractory cases warrants further investigation. Future research should explore whether KTPs can offer improved outcomes for patients whose PWBs remain resistant to or relapse following PDL therapy. Additionally, studies should examine the laser's effectiveness in treating PWBs in infants and children.

The PDL has long been regarded as the gold standard for treating PWBs. However, since its introduction, there have been minimal advancements in laser technologies designed specifically for PWB treatment in clinical practice. In Germany, accessibility issues have been compounded by inadequate reimbursement from health insurance providers, high maintenance costs, and operational challenges associated with PDL devices and their consumables [7]. These barriers have further limited the availability of effective treatments for PWBs in the healthcare system [7].

Earlier generations of KTPs were limited by their small spot size, limiting penetration depth and reducing their effectiveness in targeting deeper dermal vessels in PWBs. Recent advancements have addressed these limitations, offering spot sizes up to 16 mm and variable sequential pulse modes (single, submillisecond, and submicrosecond) with pulse durations ranging from 0.3 to 40 ms.

While the 532 nm wavelength of the KTP has a higher absorption coefficient for melanin compared to the 585 or 595 nm wavelengths used in PDLs, the cryogen cooling system with pre‐ and post‐cooling effectively minimizes the risks of burns and PIH. In this study, no severe or long‐term AEs were observed, underscoring the improved safety profile of this approach. Other prospective clinical studies have also shown similar levels of efficacy, safety, and patient satisfaction with both laser systems [14, 15]. In summary, the KTP laser demonstrated comparable effectiveness and safety to PDL in treating PWB while offering greater stability and cost efficiency. However, due to its novelty, further studies are needed to assess long‐term outcomes.

The study has certain limitations. Its retrospective design limits the strength of its conclusions. Additionally, the absence of a control arm restricts direct comparisons with other treatment modalities. Prospective, comparative studies with a longer period and a larger cohort are necessary to validate these results. Furthermore, the majority of patients in this study had Fitzpatrick skin type II, which carries a lower risk of PIH. Given the higher risks of PIH with 532 nm lasers in Fitzpatrick skin types IV–VI, further studies are needed to evaluate safety and efficacy across a broader range of skin types.

Looking ahead, additional research is essential to refine treatment protocols and optimize outcomes. Since this KTP has only recently been introduced into clinical practice, further exploration of optimal laser settings is crucial. These efforts will enable clinicians to personalize treatments more effectively, enhancing both efficacy and predictability in PWB management.

## Author Contributions

**Lynhda Nguyen:** data curation, statistical analysis, data interpretation, manuscript writing. **Stefan W. Schneider:** data interpretation. **Katharina Herberger:** data interpretation, manuscript writing. All authors discussed the results and contributed to the final manuscript.

## Ethics Statement

The patients in this manuscript have given written informed consent to the publication of their case details.

## Conflicts of Interest

Lynhda Nguyen and Katharina Herberger have received lecture fees from Cynosure Lutronic. Stefan W. Schneider has none to be declared.

## Supporting information


Data S1.

## Data Availability

The data that support the findings of this study are available from the corresponding author upon reasonable request.
